# Supplementary material for: Challenges and Achievements in the In Vitro Culture of Balantioides coli: Insights into the Excystation Process
Source: Pathogens. 2025 Jul 23;14(8):725. doi: 10.3390/pathogens14080725 (PMC12389738; doi:10.3390/pathogens14080725)

Supplementary Figure S1. Chromatogram derived from the PCR products of the *Balantioides coli* isolates used in this study. Vertical lines demarcate distinct gene regions: the 3′ terminus of the SSU rDNA, the ITS1–5.8S–ITS2 region, and the 5′ terminus of the LSU rDNA. Heterogeneous base calls are annotated accordingly: the predominant base is shown on the primary sequence line, with alternative base calls from the secondary sequence presented on a subordinate line.

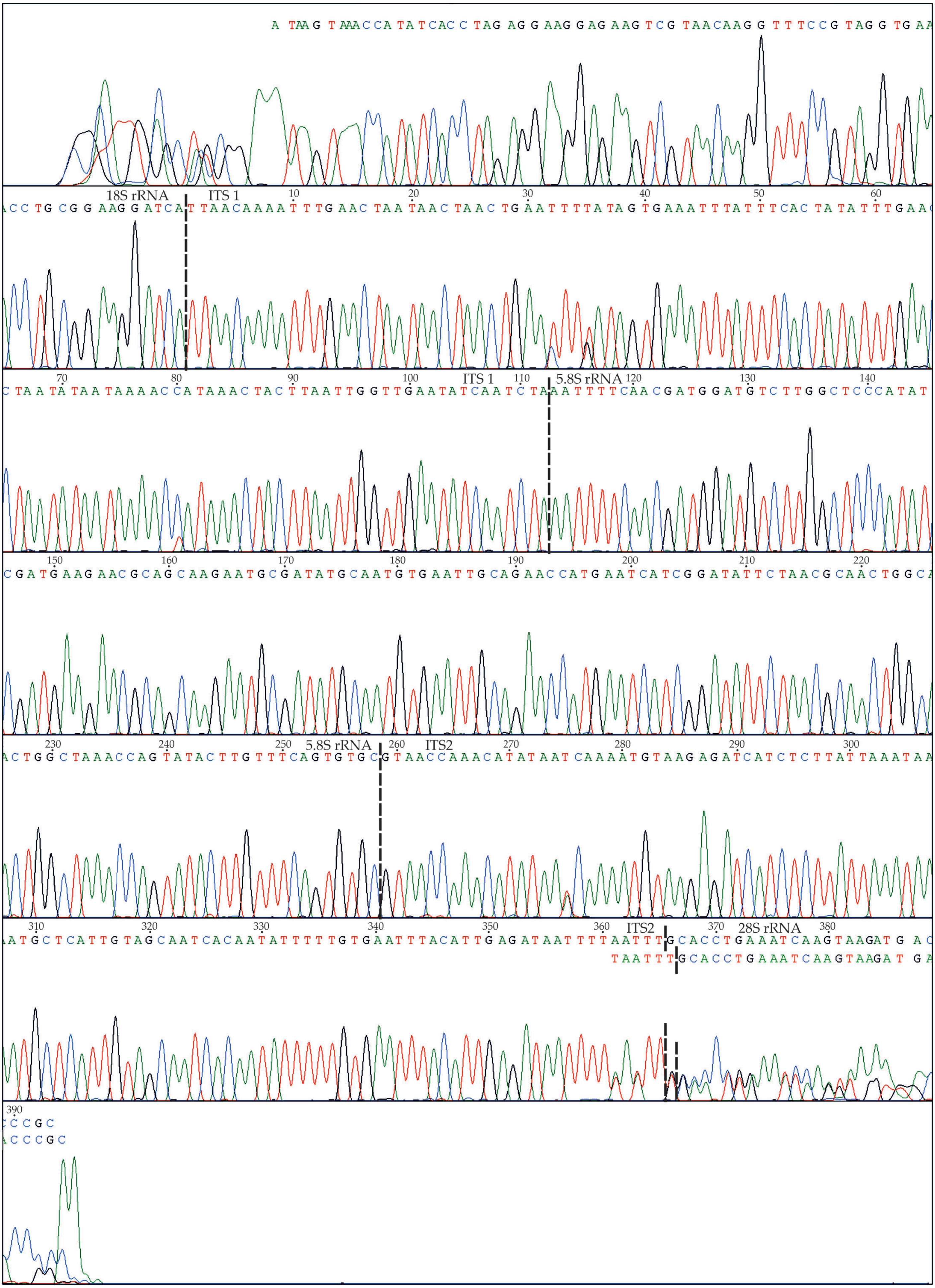

Supplement: Supplementary file 1 [file pathogens-14-00725-s001.zip › Sup. Figure S1.pdf]
